# 24-epibrassinolide stimulates imidacloprid detoxification by modulating the gene expression of *Brassica juncea* L

**DOI:** 10.1186/s12870-017-1003-9

**Published:** 2017-02-28

**Authors:** Anket Sharma, Sharad Thakur, Vinod Kumar, Anup Kumar Kesavan, Ashwani Kumar Thukral, Renu Bhardwaj

**Affiliations:** 1 Plant Stress Physiology Lab, Department of Botanical and Environmental Sciences, Guru Nanak Dev University, Amritsar, India 143005; 2 Department of Molecular Biology and Biochemistry, Guru Nanak Dev University, Amritsar, India 143005

**Keywords:** *Brassica juncea*, Imidacloprid toxicity, Brassinosteroids, Pesticide residues

## Abstract

**Background:**

Pesticides cause oxidative stress to plants and their residues persist in plant parts, which are a major concern for the environment as well as human health. Brassinosteroids (BRs) are known to protect plants from abiotic stress conditions including pesticide toxicity. The present study demonstrated the effects of seed-soaking with 24-epibrassinolide (EBR) on physiological responses of 10-day old *Brassica juncea* seedlings grown under imidacloprid (﻿﻿IMI) toxicity.

**Results:**

In the seedlings raised from EBR-treated seeds and grown under IMI toxicity, the contents of hydrogen peroxide (H_2_O_2_) and superoxide anion (O^.^_2_^−^) were decreased, accompanied by enhanced activities of superoxide dismutase (SOD), catalase (CAT), glutathione reductase (GR), glutathione-S-transferase (GST), guaiacol peroxidase (POD) and the content of glutathione (GSH). As compared to controls, the gene expressions of *SOD, CAT, GR, POD, NADH* (NADH-ubiquinone oxidoreductase)*, CXE* (carboxylesterase)*, GSH-S* (glutathione synthase)*, GSH-T* (glutathione transporter-1)*, P450* (cytochrome P450 monooxygenase) and *GST1-3,5-6* were enhanced in the seedlings raised from EBR-treated seeds and grown in IMI supplemented substratum. However, expression of *RBO* (respiratory burst oxidase, the gene responsible for H_2_O_2_ production) was decreased in seedlings raised from EBR treated seeds and grown under IMI toxicity. Further, the EBR seed treatment decreased IMI residues by more than 38% in *B. juncea* seedlings.

**Conclusions:**

The present study revealed that EBR seed soaking can efficiently reduce oxidative stress and IMI residues by modulating the gene expression of *B. juncea* under IMI stress. In conclusion, exogenous EBR application can protect plants from pesticide phytotoxicity.

## Background

Plants are susceptible to attack by various insect pests like termites, soil bugs, aphids and leaf hoppers. Pesticides are widely utilized to control these insect pests, but these pesticides cause aerial pollution upon spraying due to their volatile nature [1]. Imidacloprid (IMI) is a neonicotinoid insecticide, which is systemic in action and applied via soil/seed treatment to protect plants from soil and aerial insect pests, without causing any aerial pesticide pollution [2]. Application of pesticides causes oxidative stress to plants by generating reactive oxygen species [3, 4]. Pesticides persist in the parts of plants in form of pesticide residues [4, 5] which cause a major threat to humans as well as pollinators, including honeybees [6]. Due to their persistence in form of residues, pesticides also enter the ecosystem via the food chain, hence cause serious threat to ecosystems and the environment [1]. Plants are able to degrade pesticides into soluble and less toxic metabolites through a three-phased enzyme-mediated degradation process [7, 8]. The first phase involves enzymes like cytochrome P450 monooxygenases (P450), peroxidases and carboxylesterases, which are involved in the activation of pesticides. In the 2^nd^ phase, glutathione-S-transferase (GST) and UDP-glycosyltransferase help in the conjugation of activated pesticides with glutathione (GSH) and glucose, resulting in the formation of less toxic and more soluble metabolites. These metabolites are finally stored in vacuoles or in the apoplast in the 3^rd^ phase of pesticide detoxification. Brassinosteroids (BRs), a class of plant hormones, are well known to protect plants from abiotic stresses including that caused by the pesticides [9–12]. BRs also decrease oxidative stress in plants caused by pesticides, accompanied by enhanced activities of antioxidative enzymes [3] and a decrease of pesticide residues in plants [5, 6]. Exogenous application of BRs have been reported to enhance the expression of genes encoding P450, GST, superoxide dismutase (SOD), ascorbate peroxidase (APOX), catalase (CAT), glutathione reductase (GR) and GSH synthase, resulting in pesticide detoxification [3, 5]. Pesticide application causes oxidative stress to plants by producing reactive oxygen species (ROS), and persists in the form of pesticide residues. Antioxidative and pesticide detoxification enzymes help in scavenging ROS and reduction of pesticide residues. Keeping in mind the roles of BRs in boosting the antioxidative defence system of plants under pesticide toxicity, the present work was carried out to understand the 24-epibrassinolide (EBR)﻿-regulated pesticide detoxification mechanism in *Brassica juncea* seedlings. In earlier studies, researchers have mostly applied BRs via foliar mode, but the present study was undertaken to access the effects of seed-soaking with EBR on oxidative stress and IMI residues in 10-day old *B. juncea* seedlings grown under IMI toxicity.

## Methods

### Plant germination

Seeds of *B. juncea* L. variety RLC-1 were soaked in 0 or 100 nM EBR L^−1^ for 8 h. IMI concentrations (0, 150, 200 and 250 mg IMI L^−1^) were prepared by dissolving IMI in distilled water. 3 mL of IMI solution were poured into each Petri-plate lined with Whatman#1 filter-paper. The EBR-soaked seeds of *B. juncea* were germinated in Petri-plates containing IMI solutions and kept in a seed germinator (25 ± 0.5 °C temperature, 16 h photoperiod, and 175 μmol m^−2^ s^−1^ light intensity). Seedlings were harvested after 10 days of sowing and analysed for contents of ROS and GSH, the activities of antioxidative enzymes, expression of genes and IMI residues. All the experiments were performed in triplicates. Each replicate consisted of one Petri-plate, and 10 seedlings were randomly selected from it.

### Estimation of reactive oxygen species

#### Superoxide anions (O﻿^.^_2_^−^)

The superoxide anion content was estimated according to Wu et al. [13]. One g of plant tissue was homogenized in 6 mL of phosphate buffer (65 mM, pH = 7.8) containing 1% of polyvinylpyrrolidone. The homogenate was centrifuged at 5000 × g for 15 min at 4 °C. To 0.5 mL of supernatant, 0.5 mL of phosphate buffer (65 mM, pH = 7.8) and 0.1 mL of hydroxylamine hydrochloride (10 mM) were added. The mixture was incubated at 25 °C for 30 min. After incubation, 1 mL of 3-aminobenzenesulphonic acid (58 mM) and 1 mL of 1-naphthylamine (7 mM) were added to the mixture, followed by an incubation at 25 °C for 20 min. The absorbance was measured at 530 nm. To calculate the superoxide content, a standard curve of sodium nitrite was used and content was expressed as μmol g^−1^ FW of seedlings.

#### Hydrogen peroxide (H_2_O_2_)

H_2_O_2_ was evaluated using method given by Patterson et al. [14]. Plant tissue (0.5 g) was crushed in 1 mL of acetone, followed by centrifugation at 5000 × g for 15 min at 4 °C. To the supernatant, 20 μL of 20% titanium chloride in concentrated HCl were added. Then 200 μL of ammonia solution (17 M) were added, followed by repeated washing of the precipitate with acetone. Washed precipitates were dissolved in 1.5 mL of H_2_SO_4_ (2 N). Absorbance was read at 410 nm. The content of hydrogen peroxide was calculated from a standard curve of H_2_O_2_ and was expressed as μmol g^−1^ FW of seedlings.

### Estimation of antioxidative enzymes and glutathione content

#### Superoxide dismutase (SOD)

SOD activity was estimated according to Kono [15] with minor modifications. One g of plant tissue was homogenized in 3 mL of sodium carbonate buffer, followed by centrifugation at 12,000 × g at 4 °C for 20 min. Supernatant was used as sample for further analysis. The reaction mixture consisted of 1630 μL of sodium carbonate buffer (pH = 10.2), 500 μL of nitroblue tetrazolium (24 μM), 100 μL of EDTA (0.1 mM), 100 μL of hydroxylamine hydrochloride (1 mM), 100 μL of Triton-X-100 (0.03%) and 70 μL of sample. The absorbance was measured at 560 nm.

#### Catalase (CAT)

CAT activity was estimated according to Aebi [16] with slight modifications. 3 mL of 100 mM potassium phosphate buffer (PPB) with pH 7.0 were used for homogenization of 1 g of fresh seedlings. The homogenate was then centrifuged at 2000 × g for 20 min at 4 °C, and the supernatant was used to estimate the CAT activity. In a cuvette, the reaction mixture consisted of 1500 μL of PPB (pH = 7.0, 50 mM), 930 μL of hydrogen peroxide (15 mM) and 70 μL of sample. The absorbance was measured at 240 nm.

#### Guaiacol peroxidase (POD)

The activity of POD was determined using method given by Putter [17]. One g of seedlings was homogenized in 3 mL of PPB (100 mM, pH = 7.0) buffer, followed by centrifugation at 12,000 × g at 4 °C for 20 min. The supernatant was used as sample for further analysis. The absorbance was recorded at 436 nm by preparing a reaction mixture containing 70 μL sample with 2350 μL PPB (50 mM, pH 7.0), 50 μL guaiacol solution (20 mM) and 30 μL H2O2 (12 mM).

#### Glutathione reductase (GR)

GR activity was determined according to Carlberg and Mannervik [18]. One g of fresh seedlings was homogenized in 3 mL of PPB (100 mM, pH = 7.0) buffer followed by centrifugation at 12,000 × g at 4 °C for 20 min. The supernatant was used as plant sample to determine GR activity. The reaction mixture contained 1530 μL PPB (50 mM, pH 7.0), 300 μL each of ethylenediaminetetraacetate (EDTA) (3.0 mM), NADPH (0.1 mM), oxidized glutathione (1.0 M), and 70 μL sample. The absorbance was recorded at 340 nm.

#### Glutathione-S-transferase (GST)

The glutathione-S-transferase activity was quantified based on Habig and Jacoby [19]. One g of fresh seedlings was homogenized in 3 mL of PPB (100 mM, pH = 7.5) buffer and centrifuged at 12,000 × g at 4 °C for 20 min. The supernatant was used as sample in reaction mixture. The reaction mixture contained 70 μL sample, 1930 μL PPB (50 mM, pH 7.5), and 250 μL each of reduced glutathione (10 mM) and 1-chloro-2,4-dinitrobenzene (10 mM). The absorbance was measured at 340 nm.

#### Glutathione (GSH)

The glutathione content was determined according to the scheme given by Sedlak and Lindsay [20]. Fresh plant tissue (1 g) was homogenized in 3 mL of Tris buffer (50 mM, pH 10.0) containing 1 mM EDTA. The homogenate was then subjected to centrifugation at 12,000 × g for 15 min, and the supernatant from the plant extract was used to estimate the GSH content. To the 100 μL of plant extract, 1 mL of Tris buffer, 50 μL of Ellman’s reagent and 4 mL of absolute methanol were added, and kept at room temperature for 15 min and then subjected to centrifugation at 3000 × g for 15 min. The absorbance of the supernatant was measured noted at 412 nm.

### Gene expression analysis

Total RNA was extracted from whole seedlings using Trizol method according to the manufacturer’s instructions (Invitrogen). Total RNA was used for reverse transcription with an RNA to cDNA kit (Invitrogen), containing MuLV as reverse transcriptase, dNTP’s mix, random octamers and oligo (dT)_16_. Gene-specific primers (Table 1) were designed according to the mRNA sequence from Genbank and EMBL database, and the *actin* gene was used as an internal control due to its high expression stability in the vegetative stage of plants. Quantitative real time PCR (qRT-PCR) was performed using the StepOne™ real time detection system (Applied Biosystems) and *Power* SYBR® Green PCR Master Mix (three biological and technical replicates). A melting curve was generated at the end of the each PCR cycle, which verified that a single product was amplified, using the software provided along with the PCR system. The mRNA quantification was based on the method of Livak and Schmittgen [21]. ΔCt values were obtained by subtracting the threshold value (Ct) of the internal control (*actin*) from that of the gene of interest. ΔΔCt values were obtained by subtracting the Ct values of the untreated control sample from the ΔCt value. The fold-changes in the expression levels relative to the untreated samples were expressed as 2^-ΔΔCt^.

**Table 1:** Primers used for quantitative real time polymerase chain reaction (qRT-PCR)

Gene name	Primer sequence
*actin*	Forward primer 5′ CTTGCACCTAGCAGCATGAA 3′ Reverse primer 5′ GGACAATGGATGGACCTGAC 3′
*SOD*	Forward primer 5′GGTTTCCATGTCCATGCTCT 3′ Reverse primer 5′ATTGTGAAGGTGGCAGTTCC 3′
*CAT*	Forward primer 5′ TCAGCTGCCAGTTAATGCAC 3′ Reverse primer 5′ GACAGCAGGTGGAGTTGGAT 3′
*GR*	Forward primer 5′ AAGGCAAAAGAAGGTGCTGA 3′ Reverse primer 5′ AGTTCCCTTGCTGGTCTTCA 3′
*RBOH*	Forward primer 5′ACGGGGTGTGATAGAGATGC 3′ Reverse primer 5′TTTTTCCAGTTGGGTCTTGC 3′
*NADH*	Forward primer 5′CTCGGCCTTTCTCAACAGAC 3′ Reverse primer 5′CATTTCCCAAGTTTCCCAGA 3′
*CXE*	Forward primer 5′ GGCGCTAACATGACTCATCA 3′ Reverse primer 5′ CTCCCAGAGTTGAGCGATTC 3′
*GSH-S*	Forward primer 5′ CCCATCTTCAACGAGTTGGT 3′ Reverse primer 5′ GTGCAAACCCAAACGAATCT 3′
*GSH-T*	Forward primer 5′GCTGGTCACAGGAACCATCT 3′ Reverse primer 5′CTACTTCAGTGCCCCACCAT 3′
*GST-1*	Forward primer 5′CGTCGTCGAAGAAGAAGAGG 3′ Reverse primer 5′TTTTTGGTGGGAGTTCCAAG 3′
*GST-2*	Forward primer 5′AGACCAAGCCGTTGTTGAAG 3′ Reverse primer 5′TTTTTGGTGGGAGTTCCAAG 3′
*GST-3*	Forward primer 5′TACGAGGCTAGGCTCAAGGA 3′ Reverse primer 5′AGCCACCCACTCGTTAACAC 3′
*GST-4*	Forward primer 5′CAAGGAACCAACCTTCTCCA 3′ Reverse primer 5′TGGTCAGTGGTCAAGCCATA 3′
*GST-5*	Forward primer 5′AGTGGCTGCAAAGCTTGTTT 3′ Reverse primer 5′TGTGGTGAAGATCGGTCAAA 3′
*GST-6*	Forward primer 5′GCCGAAGAGGAGGCTAAGTT 3′ Reverse primer 5′TCGGTGAAGAGCTTCTTGGT 3′
*POD*	Forward primer 5′ TTCGAACGGAAAAAGATGCT 3′ Reverse primer 5′ AACCCTCCATGAAGGACCTC 3′
*P450*	Forward primer 5′ CATTTGTTCTCACCCACACG 3′ Reverse primer 5′ CACAACCGAGTTCGTGAATG 3′

### Analysis of IMI residues

AOAC, the official method 2007.01 [22] was followed to prepare seedling extracts for IMI residue analysis. One g of seedlings was crushed in 1 mL 1% acetic acid in acetonitrile, and 200 mg of anhydrous MgSO_4_ and 50 mg sodium acetate were added. The mixture was shaken for 2 min, followed by centrifugation at 1500 × g for 5 min. 0.5 mL of the upper layer was taken, and 75 mg anhydrous MgSO_4_ along with 25 mg of primary secondary amine (PSA) sorbent were added. The mixture was again centrifuged, followed by filtration of the upper layer with 0.22 μ filters and stored at 4 °C until analysis.

#### GC-MS analysis

GC-MS (QP2010 Plus, Shimadzu, Kyoto, Japan) was used to analyse the seedling extracts for IMI residues. Helium was used as carrier gas, the initial oven column temperature was set at 50 °C, followed by increasing it to 125 °C at 25 °C min^−1^ and finally increased to 300 °C at 10 °C min^−1^ (hold for 15 min). The sample injector temperature was set to 250 °C, the injection mode was split, column flow rate was 1.70 mL min^−1^, the analytical column used was DB-5 ms. The ion source and interface temperatures were set to 200 °C and 280 °C respectively. The volume of the sample injected was 8 μL.

### Statistical analysis

The results were analysed using Two-way ANOVA, Tukey’s HSD and multiple linear regression analysis (MLR), using self-coded software (MS-Excel 2010) and artificial neural networks (ANN), using Statistica-12. In ANN model, contents of oxidative stress markers and GSH, activities of antioxidative enzymes, relative expression of genes and IMI residues (targets) were regressed against the concentration of applied IMI and EBR (inputs), using 3 neurons, 1 hidden layer, *tanh* function from input to neurons, and from neurons to output.

## Results

### Effects of EBR on ROS

Imidacloprid (IMI) application was observed to increase the oxidative stress in *B. juncea* seedlings by enhancing the contents of H_2_O_2_ and O^.^_2_^−^. However, seed soaking with 100 nM EBR resulted in decreasing the contents of these reactive oxygen species (ROS) in the seedlings grown in IMI supplemented Petri-plates (Table 2). Statistical analysis of data using two-way ANOVA and Tukey’s HSD revealed that there was a significant difference in ROS contents in the seedlings of *B. juncea* plants. Multiple linear regression (MLR) analysis revealed that the partial regressions between the concentrations of EBR used for seed soaking and the contents of H_2_O_2_ and O^.^_2_^−^ generated were regressed negatively, whereas IMI regressed positively on the generation of these ROS. This indicated the role of EBR in decreasing the ROS generation (Table 2).

**Table 2:** Effect of EBR seed-soaking on the contents of stress markers and glutathione along with activities of antioxidative enzymes in *Brassica juncea* seedlings grown in IMI supplemented substratum (Mean ± SD, Two-way ANOVA, Tukey’s HSD, multiple linear regression)

Treatments		H_2_O_2_ content (μmol g^−1^ FW)	O^.^_2_^−^ content (μmol g^−1^ FW)	SOD activity (Units mg^−1^ protein)	CAT activity (μmol min^−1^ mg^−1^ protein)	POD activity (μmol min^−1^ mg^−1^ protein)	GR activity (μmol min^−1^ mg^−1^ protein)	GST activity (μmol min^−1^ mg^−1^ protein)	GSH content (m﻿g g^−1^ FW)
IMI (mg L^−1^)	EBR (nM)								
0	0	12.70 ± 1.27	0.97 ± 0.15	29.27 ± 3.29	5.68 ± 0.74	35.10 ± 2.88	5.91 ± 0.24	26.48 ± 1.06	0.51 ± 0.02
0	100	11.48 ± 1.87	0.88 ± 0.12	38.85 ± 4.95	7.57 ± 0.83	52.94 ± 4.60	8.51 ± 0.59	34.20 ± 2.33	0.75 ± 0.05
150	0	15.22 ± 1.53	1.64 ± 0.29	36.25 ± 5.03	6.71 ± 0.98	44.14 ± 5.19	7.83 ± 0.60	28.99 ± 1.52	0.56 ± 0.03
150	100	10.11 ± 0.35	1.02 ± 0.20	53.98 ± 6.24	9.93 ± 1.14	71.46 ± 4.85	10.81 ± 0.66	37.47 ± 2.51	0.69 ± 0.03
200	0	17.41 ± 3.17	2.56 ± 0.52	32.85 ± 3.52	5.19 ± 0.85	68.23 ± 8.52	7.91 ± 0.41	31.25 ± 1.25	0.50 ± 0.04
200	100	10.94 ± 1.61	1.64 ± 0.17	50.72 ± 6.23	8.94 ± 0.50	94.40 ± 10.41	9.66 ± 1.20	38.67 ± 2.13	0.72 ± 0.07
250	0	21.70 ± 0.47	3.14 ± 0.60	27.98 ± 5.22	3.50 ± 0.74	43.93 ± 3.71	5.88 ± 0.64	25.71 ± 1.01	0.39 ± 0.06
250	100	14.81 ± 0.86	1.75 ± 0.29	42.67 ± 3.43	6.47 ± 0.78	74.28 ± 8.45	7.11 ± 1.03	32.89 ± 0.98	0.62 ± 0.04
F_IMI_ F_EBR_ F_IMI×EBR_ HSD (*p* < 0.05)		17.53*** 54.84*** 3.77* 4.59	25.53*** 30.32*** 3.98* 0.95	6.99** 56.66*** 0.95 13.77	16.14*** 74.60*** 1.32 2.31	33.01*** 89.59*** 0.99 18.60	19.73*** 51.55*** 1.77 2.06	14.00*** 122.45*** 0.16 4.81	10.79*** 128.84*** 2.03 0.08
Multiple linear regression equation					β-regression coefficients		Multiple correlation coefficient		Significant at *p* <
					β_IMI_	β_EBR_			
H_2_O_2_ content = 13.6710 + 0.0205 X_1_–0.0490 *X*_2_					0.4994	–0.6379	0.8102		0.001
O^.^_2_^−^ content = 1.1777 + 0.0060 X_1_–0.0080 *X*_2_					0.7037	–0.4741	0.8485		0.001
SOD activity = 29.388 + 0.0146 X_1_ + 0.1497 *X*_2_					0.1403	0.7662	0.7790		0.001
CAT activity = 5.8784–0.0041 X_1_ + 0.0296 *X*_2_					–0.1852	0.7222	0.7465		0.001
POD activity = 33.3930 + 0.0963 X_1_ + 0.2542 *X*_2_					0.4660	0.6569	0.8054		0.001
GR activity = 6.8544 + 0.0002 X_1_ + 0.0214 *X*_2_					0.0110	0.6247	0.6249		0.01
GST activity = 27.562 + 0.0036 X_1_ + 0.0770 *X*_2_					0.0725	0.8226	0.8258		0.001
GSH content = 0.5507–0.0004 X_1_ + 0.0021 *X*_2_					–0.3077	0.8384	0.8931		0.001

X_1_ = IMI (mg L^−1^), *X*_2_ = EBR (nM), *, ** and *** = significant at *p* < 0.05, *p* < 0.01 and *p* < 0.001 respectively

### Effects of EBR on Glutathione content and activities of antioxidative enzymes

The content of GSH as well as the activities of antioxidative enzymes like SOD, CAT, POD, GR and GST were enhanced in the seedlings raised from EBR-treated seeds and grown in Petri-plates supplemented with IMI solutions (Table 2). Significant differences in SOD, CAT, POD, GR and GST activities and GSH content were observed after analysing the data, using two-way ANOVA and Tukey’s HSD. MLR analysis and positive β_EBR_ regression coefficients also showed that EBR application increases the GSH content and activities of antioxidative enzymes (Table 2).

### Effects of EBR on gene expression

**Fig. 1:**
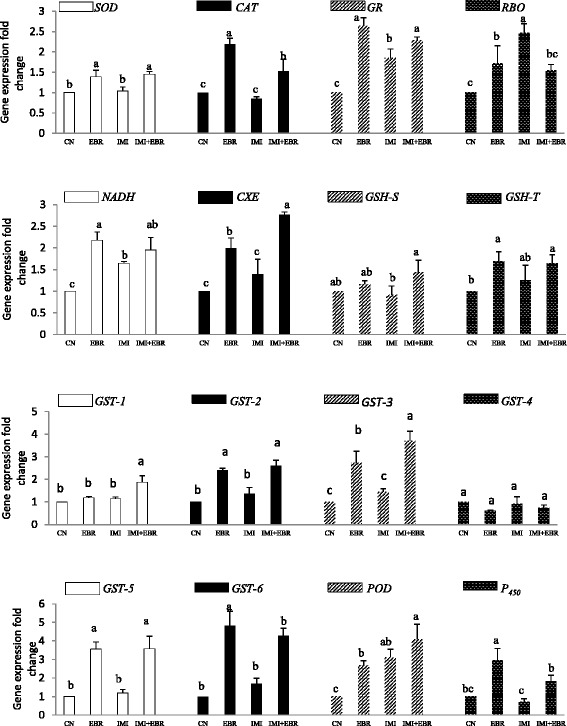
Effect of EBR seed-soaking on the relative expression of genes involved in IMI stress amelioration in *Brassica juncea* seedlings. Data are shown as the mean ± standard deviation (three biological replicates), bars with the same letters indicate no significant difference at *p* < 0.05 (comparison among treatments of same gene). HSD values for each gene have been mentioned in Table 3. (EBR concentration = 100 nM and IMI concentration = 200 mg IMI L^−1^)

We observed that seed soaking with EBR significantly modulated the gene expression in *B. juncea* seedlings grown under IMI toxicity (Fig. 1). The fold-change expression of the *RBO* gene was decreased by 37.65% in seedlings raised from EBR-treated seed, and grown under IMI toxicity, as compared to seedlings raised from untreated seeds and grown under IMI toxicity. On similar comparison of *B. juncea* seedlings, an increase in the expression of *SOD* (39.42%), *CAT* (78.82%), *GR* (23.24%), *POD* (31.51%), *GST1* (64.04%), *GST2* (90.51%), *GST3* (157.64%), *GST5* (203.39%), *GST6* (154.76%), *GSH-S* (56.52%), *GSH-T* (32.0%), *CXE* (99.28%), *NADH* (18.18%) and *P450* (152.78%) was noticed (Fig. 1). We observed a significant difference in the expression of genes encoding enzymes involved in pesticide detoxification enzymes in *B. juncea* seedlings after analysing the data using two-way ANOVA and Tukey’s HSD. Positive β_EBR_ values obtained from the MLR analysis revealed that EBR seed soaking up-regulated the expression of all the genes (encoding enzymes involved in pesticide detoxification enzymes), except *GST-4* (Table 3).

**Table 3:** Two-way ANOVA and multiple linear regression analysis (MLR) of the relative gene expression in *Brassica juncea* seedlings raised from EBR-soaked seeds and grown in IMI supplemented substratum

Gene name	F-ratios			HSD (*p* < 0.05)
	F_IMI_	F_EBR_	F_IMI×EBR_	
*SOD*	0.78	48.57***	0.01	0.26
*CAT*	16.66**	87.07***	6.89*	0.45
*GR*	7.05*	124.34***	42.83***	0.41
*RBOH*	19.06**	0.46	31.10***	0.66
*NADH*	4.16	51.72***	18.29**	0.46
*CXE*	21.64**	88.35***	2.26	0.56
*GSH-S*	0.93	10.57*	2.86	0.46
*GSH-T*	0.59	17.10**	1.14	0.60
*GST-1*	21.14**	26.40***	8.68*	0.40
*GST-2*	6.86*	143.74***	0.52	0.49
*GST-3*	12.82**	99.63***	1.85	0.90
*GST-4*	0.09	8.38*	1.07	0.43
*GST-5*	0.19	110.10***	0.11	1.06
*GST-6*	0.059	143.70***	5.28	1.21
*POD*	40.71***	23.44**	1.66	1.24
*P450*	11.03*	51.62***	4.06	0.96
MLR equation	β-regression coefficient		Multiple correlation coefficient	Significant at *p* <
	β_IMI_	β_EBR_		
*SOD* expression = 0.9960 + 0.0003 X_1_ + 0.0041 *X*_2_	0.1169	0.9201	0.9274	0.001
*CAT* expression = 1.1311–0.0020 X_1_ + 0.0093 *X*_2_	– 0.3748	0.8566	0.9351	0.001
*GR* expression = 1.3034 + 0.0012 X_1_ + 0.0103 *X*_2_	0.1968	0.8260	0.8491	0.001
*RBOH* expression = 1.4136 + 0.0032 X_1_–0.0010 *X*_2_	0.5702	– 0.0892	0.5771	0.10
*NADH* expression = 1.2212 + 0.0011 X_1_ + 0.0074 *X*_2_	0.2250	0.7933	0.8246	0.01
*CXE* expression = 0.9051 + 0.0029 X_1_ + 0.0118 *X*_2_	0.4242	0.8571	0.9563	0.001
*GSH-S* expression = 0.9113 + 0.0005 X_1_ + 0.0034 *X*_2_	0.2040	0.6875	0.7170	0.02
*GSH-T* expression = 1.0713 + 0.0005 X_1_ + 0.0055 *X*_2_	0.1483	0.7983	0.8120	0.01
*GST-1* expression = 0.8681 + 0.0021 X_1_ + 0.0046 *X*_2_	0.5737	0.6411	0.8604	0.001
*GST-2* expression = 1.0400 + 0.0015 X_1_ + 0.0133 *X*_2_	0.2077	0.9504	0.9728	0.001
*GST-3* expression = 0.8635 + 0.0036 X_1_ + 0.0200 *X*_2_	0.3238	0.9025	0.9588	0.001
*GST-4* expression = 0.9497 + 0.0002 X_1_–0.0030 *X*_2_	0.0747	– 0.6909	0.6949	0.02
*GST-5* expression = 1.0393 + 0.0005 X_1_ + 0.0248 *X*_2_	0.0401	0.9643	0.9651	0.001
*GST-6* expression = 1.3087 + 0.0003 X_1_ + 0.0322 *X*_2_	0.0195	0.9565	0.9567	0.001
*POD* expression = 1.1777 + 0.0088 X_1_ + 0.0134 *X*_2_	0.7426	0.5635	0.9322	0.001
*P450* expression = 1.2155–0.0036 X_1_ + 0.0154 *X*_2_	– 0.3842	0.8312	0.9157	0.001

X_1_ = IMI (mg L^−1^), *X*_2_ = EBR (nM), *, ** and *** = significant at *p* < 0.05, *p* < 0.01 and *p* < 0.001 respectively

### The Effect of EBR on IMI residues

**Fig. 2:**
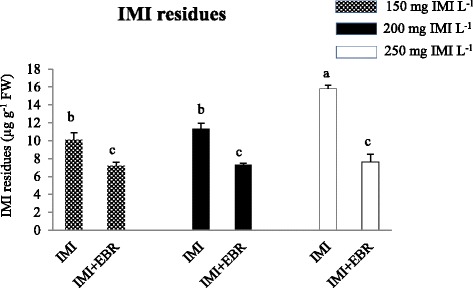
Effect of EBR seed-soaking on the IMI residues in *B. juncea* seedlings. Data are shown as the mean ± standard deviation (three biological replicates), bars with the same letters indicate no significant difference at *p* < 0.05. (EBR concentration = 100 nM)

IMI residues were reduced by 38% in the seedlings raised from EBR seed soaking and germinated in IMI-treated Petri-plates (Fig. 2). The analysis of data using two-way ANOVA and Tukey’s HSD showed a significant difference for IMI residues in *B. juncea* seedlings raised from untreated seeds and EBR treated seeds and grown under IMI toxicity. A negative β_EBR_ regression coefficient for IMI residues also revealed that seed soaking with EBR results in decreasing the IMI residues (Table 4).

**Table 4:** Two-way ANOVA and multiple linear regression analysis (MLR) of the IMI residues in *Brassica juncea* seedlings raised from EBR-soaked seeds and grown in IMI supplemented substratum

Parameter	F-ratios			HSD (*p* < 0.05)
	F_IMI_	F_EBR_	F_IMI×EBR_	
IMI residues	44.21***	322.65***	33.31***	1.62
MLR equation		β-regression coefficients		Multiple correlation coefficient
		β_IMI_	β_EBR_	
IMI residues = 6.2997 + 0.0305 X_1_ − 0.0500 *X*_2_		0.4031	–0.8117	0.9062***

X_1_ = IMI (mg L^−1^), *X*_2_ = EBR (nM), *** = significant at *p* < 0.001

Our data analysis using ANN also showed that experimental (target) and simulated (output) data were highly correlated, which indicates that ANN simulates the physiological studies carried out in the current experiment at a very high level of significance (Table 5).

**Table 5:** Data showing correlation coefficients obtained from artificial neural networks model

Parameter	Correlation coefficient		
	Train	Test	Validation
*SOD* expression	0.9255***	0.9990***	0.9178***
*CAT* expression	0.8995***	0.9681***	0.9940***
*POD* expression	0.9231***	0.9999***	0.9546***
*GST-1* expression	0.9629***	0.9987***	0.9999***
*GST-2* expression	0.9274***	0.7158***	0.9653***
*GST-3* expression	0.9632***	0.9866***	0.9629***
*GST-4* expression	0.9688***	0.9993***	0.9904***
*GST-5* expression	0.7949***	0.4758**	0.9498***
*GST-6* expression	0.9696***	0.9988***	0.9976***
*GR* expression	0.9769***	0.9936***	0.9835***
*CXE* expression	0.9833***	0.9868***	0.9846***
*P450* expression	0.9421***	0.9603***	0.9873***
*NADH* expression	0.9593***	0.9992***	0.9243***
*RBO* expression	0.9469***	0.9966***	0.9997***
*GSH-T* expression	0.9555***	0.9984***	0.9295***
*GSH-S* expression	0.8435***	0.9260***	0.9822***
H_2_O_2_ content	0.9053***	0.8859***	0.9317***
O_2_^−^ content	0.9017***	0.9784***	0.9379***
GSH content	0.8829***	0.8760***	0.9166***
SOD activity	0.8899***	0.9082***	0.9565***
CAT activity	0.9193***	0.9275***	0.9615***
POD activity	0.8475***	0.9081***	0.8491***
GST activity	0.9396***	0.9401***	0.9090***
GR activity	0.9235***	0.9269***	0.9573***
IMI residues	0.9890***	0.9809***	0.9766***

** and *** = significant at *p* < 0.01 and *p* < 0.001 respectively

## Discussion

In the present investigation, the contents of ROS including and O^.^_2_^−^ and H_2_O_2_ were noticed to increase with the application of IMI. A decrease in the contents of these ROS was noticed after EBR seed treatment. One of the reasons for the production of these ROS might be oxidative burst caused by abiotic stress conditions due to the disruption of the antioxidative defense system [23]. It has also been demonstrated that *RBOH1* (Respiratory burst oxidase homologue1) is responsible for the production of H_2_O_2_ in plants under pesticide stress [4]. Moreover, in the current study, we also observed that the expression of the *RBO* gene (respiratory burst oxidase, a gene which is responsible for the production of H_2_O_2_) was up-regulated in *B. juncea* plants grown under IMI toxicity. It has been observed that the O_2_^.-^ content was decreased after seed application of EBR, and it might have occurred as a result of the conversion of O_2_^.-^ to H_2_O_2_ by superoxide dismutase (SOD), whose activity as well as gene expression (*SOD)* in the current experiment was also observed to be enhanced after the seed treatment of EBR. Additionally, the content of H_2_O_2_ was also noticed to decline with seed application of EBR, and the possible reason behind this could be the conversion of H_2_O_2_ to water and molecular oxygen by the antioxidative enzyme catalase (CAT). In the current study, the activity of the CAT enzyme and the expression of the *CAT* gene were also increased in seedlings raised from EBR-soaked seeds and grown in Petri-plates containing IMI. Moreover, the expression of *RBO* has also been observed to decrease with the EBR application, suggesting another reason for the significant reduction of H_2_O_2_. Studies carried out by Hayat et al. [24] and Fariduddin et al. [25] also showed that BRs play an important role in the scavenging of ROS in plants under environmental stress conditions.

As a result of oxidative stress caused by pesticide toxicity, the antioxidative defense system gets activated in order to efficiently scavenge the ROS and ultimately reduce the oxidative stress [3]. In the current study, the GSH content and the activities of enzymatic antioxidants including SOD, CAT, POD, GR and GST were observed to increase with IMI toxicity (except higher IMI concentration) as well as with the EBR seed application. SOD and CAT are involved in the conversion of harmful superoxide anions to non-toxic water and molecular oxygen. Additionally, it has also been reported that another pathway, the ascorbate-glutathione cycle, is involved in the detoxification of H_2_O_2_ [26]. Glutathione is involved in reduction of H_2_O_2_ into water by the ascorbate-glutathione cycle, which is also catalyzed by the GR enzyme [27]. In the ascorbate-glutathione cycle, GR also plays an important role in reduction of oxidative stress by maintaining the ratio of reduced and oxidized glutathione. In the current study, specific activity of GR was also observed to increase with the application of IMI, which suggests that GR is actively involved in detoxification of ROS generated as a result of IMI toxicity. Similar increase in GR activity was observed in *Vigna radiata* plants grown under chlorpyrifos toxicity [28]. EBR seed application in the current study further enhanced the activities of all these antioxidative enzymes under IMI toxicity. The alterations of the activities of these antioxidative enzymes might be due to the EBR modulated protein synthesis or altered enzyme kinetics [29, 30]. Moreover, in the current study, we noticed that gene expression of *SOD*, *CAT*, *GR*, *POD* and *GST1-3,5-6* was also up-regulated in seedlings raised from EBR-treated seeds and grown under IMI toxicity. This suggests that the increase in the expression of the genes encoding these antioxidative enzymes might be one of the reasons for enhanced specific activities of antioxidative enzymes in *B. juncea* plants grown from EBR-treated seeds and grown under IMI stress.

In the current study, IMI residues were observed to decrease in seedlings raised from EBR-treated seeds and grown under IMI toxicity. As described in the introduction section, the three phased enzyme-mediated detoxification system is responsible for pesticide degradation in plants [7, 8]. In the current study, activities of POD and GST enzymes, which are involved in three phased pesticide detoxification system, were observed to increase with the IMI application as well as EBR seed treatment. Additionally, the gene expression of phase-1 enzyme *viz., P450, POD, CXE* and phase-2 enzyme *GST* along with the *GSH-S* and *GSH-T* was also observed to increase with EBR application under IMI toxicity. Oxidoreductase helps in pesticide detoxification [4] and the gene expression of *NADH* was also noticed to enhance after EBR seed application under IMI toxicity. Since the expression of genes (encoding enzymes involved in pesticide detoxification system) was modulated by EBR-seed soaking, this could be the possible reason for the reduction of IMI residues in *B. juncea* seedlings raised from EBR-treated seeds and grown under IMI toxicity.

## Conclusions

The current study demonstrates that seed soaking with EBR enhances IMI detoxification and decreases oxidative stress in *B. juncea* seedlings through the up-regulation of *SOD, CAT, GR, POD, NADH, CXE, GSH-S, GSH-T, P450* and *GST1-3,5-6* genes involved in enzymatic pesticide detoxification accompanied by down-regulation of *RBO* gene. As compared to earlier studies that were based on foliar application of BRs, the important point in the current study is that a single application of EBR via seed treatment can efficiently activate the plant defence system against pesticide stress by modulation of gene expression.

## Abbreviations

ANOVA: Analysis of variance
AOAC: Association of official analytical chemists
cDNA: Complimentary deoxyribonucleic acid
EBR: 24-epibrassionolide
EMBL: European molecular biology laboratory
FW: Fresh weight
HSD: Honestly significant difference
MLR: Multiple linear regression
PCR: Polymerase chain reaction
PSA: Primary secondary amine
RNA: Ribonucleic acid
